# Genome wide association study to identify SNPs associated with Homocysteine, Vitamin B_12_ and holotranscobalamin in Indian Population

**DOI:** 10.1186/1755-8166-7-S1-P51

**Published:** 2014-01-21

**Authors:** Vinay Singh Tanwar, Gaurav Garg, Ankur Mukherjee, Ganesan Karthikeyan, Sandeep Seth, Analabha Basu, Shantanu Sengupta

**Affiliations:** 1 CSIR-Institute of Genomics and Integrative Biology, Delhi, India; 2 National Institute of Biomedical Genomics, Kalyani, West Bengal, India; 3 Department of Cardiology, All India Institute of Medical Sciences, New Delhi, India

## Background

Vitamin B_12_, a cofactor for the enzyme methionine synthase, catalyzes the remethylation of homocysteine to methionine. About 50-60% of the Indian population are deficient in vitamin B_12_, a micronutrient that is only synthesized by microorganisms while mammals have evolved ways for its absorption from diet. Of the various factors involved in Vitamin B_12_ absorption, Transcobalamin II (TC II) is most important as vitamin B_12_ bound to TCII is bioavailable. Thus, the objective of our study was to identify the genetic variants that are associated with homocysteine, vitamin B_12_ and holotranscobalamin levels.

## Methods

A total of 3024 healthy individuals of Indo-European ethnicity were included in the study. Biochemical parameters like homocysteine, vitamin B_12_, holotranscobalamin etc were determined for each individual. In the first phase (discovery phase) Genome wide association studies were performed using Illumina Omni express chip and 524 individuals were genotyped for 731,442 single nucleotide polymorphisms (SNPs). Statistical analysis was performed using PLINK software (v 1.07) after stringent quality control. In the second phase SNPs that were found to be significantly associated were genotyped in 2500 healthy individuals.

## Results

Several genetic variants, some of which are novel, were found to be significantly associated with homocysteine, vitamin B_12_ and holotranscobalamin. This is the first GWAS for holoTC which we found is a better predictor of vitamin B12 status.

## Conclusion

Although we found several SNPs earlier reported to be significantly associated with the biochemical traits measured in our population also, many of the SNPs were previously not reported to be associated with the biochemical traits measured.

